# CD33 BiTE^®^ molecule-mediated immune synapse formation and subsequent T-cell activation is determined by the expression profile of activating and inhibitory checkpoint molecules on AML cells

**DOI:** 10.1007/s00262-023-03439-x

**Published:** 2023-04-11

**Authors:** Anetta Marcinek, Bettina Brauchle, Lisa Rohrbacher, Gerulf Hänel, Nora Philipp, Florian Märkl, Thaddäus Strzalkowski, Sonja M. Lacher, Dragica Udiljak, Karsten Spiekermann, Sebastian Theurich, Sebastian Kobold, Roman Kischel, John R. James, Veit L. Bücklein, Marion Subklewe

**Affiliations:** 1 Department of Medicine III, University Hospital, LMU Munich, Munich, Germany; 2 Laboratory for Translational Cancer Immunology, LMU Gene Center, Munich, Germany; 3 German Cancer Consortium (DKTK) and German Cancer Research Center (DKFZ), Heidelberg, Germany; 4 Experimental Leukemia and Lymphoma Research (ELLF), Department of Medicine III, University Hospital, LMU Munich, Munich, Germany; 5 Cancer-and Immunometabolism Research Group, LMU Gene Center, Munich, Germany; 6 Division of Clinical Pharmacology, Department of Medicine IV; Member of the German Center for Lung Research (DZL), University Hospital, LMU, Munich, Germany; 7 AMGEN Research Munich GmbH, Munich, Germany; 8 AMGEN Inc., Thousand Oaks, CA USA; 9 Division of Biomedical Sciences, Warwick Medical School, University of Warwick, Coventry, UK

**Keywords:** Acute myeloid leukemia, Bispecific antibodies, Immune synapse, Costimulation (CD86), Checkpoint molecule (PD-L1)

## Abstract

Bispecific T-cell engager (BiTE^®^) molecules recruit T cells to cancer cells through CD3ε binding, independently of T-cell receptor (TCR) specificity. Whereas physiological T-cell activation is dependent on signal 1 (TCR engagement) and signal 2 (co-stimulation), BiTE molecule-mediated T-cell activation occurs without additional co-stimulation. As co-stimulatory and inhibitory molecules modulate the strength and nature of T-cell responses, we studied the impact of the expression profile of those molecules on target cells for BiTE molecule-mediated T-cell activation in the context of acute myeloid leukemia (AML). Accordingly, we created a novel in vitro model system using murine Ba/F3 cells transduced with human CD33 ± CD86 ± PD-L1. T-cell fitness was assessed by T-cell function assays in co-cultures and immune synapse formation by applying a CD33 BiTE molecule (AMG 330). Using our cell-based model platform, we found that the expression of positive co-stimulatory molecules on target cells markedly enhanced BiTE molecule-mediated T-cell activation. The initiation and stability of the immune synapse between T cells and target cells were significantly increased through the expression of CD86 on target cells. By contrast, the co-inhibitory molecule PD-L1 impaired the stability of BiTE molecule-induced immune synapses and subsequent T-cell responses. We validated our findings in primary T-cell-AML co-cultures, demonstrating a PD-L1-mediated reduction in redirected T-cell activation. The addition of the immunomodulatory drug (IMiD) lenalidomide to co-cultures led to stabilization of immune synapses and improved subsequent T-cell responses. We conclude that target cells modulate CD33 BiTE molecule-dependent T-cell activation and hence, combinatorial strategies might contribute to enhanced efficacy.

**Supplementary Information:**

The online version contains supplementary material available at 10.1007/s00262-023-03439-x.

## Introduction

Within the last 5 years, several novel agents have been approved for use against acute myeloid leukemia (AML), changing the treatment landscape [1–3]. Despite these advances, overall survival rate is poor and high relapse rates remain a challenge. Nevertheless, allogeneic stem cell transplantation is still the most potent anti-leukemic treatment strategy, with donor lymphocyte infusions demonstrating the power of T cells to eliminate chemo-refractory AML cells [4].

The success of blinatumomab, a CD19xCD3 bispecific T-cell-engager (BiTE^®^) molecule for treating relapsed/refractory (r/r) and minimal residual disease-positive (MRD^+^) B-cell precursor acute lymphoblastic leukemia (BCP-ALL), has shown that alternative T-cell-recruiting strategies are also able to eliminate leukemias [5, 6]. The results of further trials utilizing CD20-directed bispecific antibodies in mature B-cell malignancies underline the potential of T-cell engagers in combating hematological malignancies [7, 8]. Translating this success to AML has proved to be challenging, and the response rates reported to date have not met expectations. Currently, various formats of T-cell-redirecting antibodies against tumor-associated antigens in AML are undergoing (pre-)clinical investigation. These investigations include phase I/II trials with a WT-1 T-cell bispecific antibody (RO7283420) and studies on CD3-engaging BiTE molecules directed against CD33 (CD33*CD3 BsAb and AMG 330) and CD135/FLT-3 (CLN-049) (NCT04580121, NCT05077423, NCT02520427, NCT05143996, respectively) [9–16].

Bispecific T-cell antibodies recruit T cells against cancer cells only through CD3ε binding and independently of T-cell receptor (TCR) specificity and expression of major histocompatibility complex molecules [17]. In contrast to our physiological understanding of T-cell activation, which is dependent on signal 1 (TCR engagement) and signal 2 (co-stimulation), BiTE molecule-mediated T-cell triggering is devoid of extrinsic co-stimulation. Previous studies have shown that T-cell co-signaling ligands on target cells, such as CD80, CD86 and PD-L1, modulate the function and activity of T cells redirected by bispecific T-cell antibodies in vitro [18–20]. However, their relevance to the formation and stabilization of immune synapses (the interface between two conjugating cells) induced by BiTE molecules is poorly understood. In physiological antigen-presenting cell (APC)–T-cell conjugates, immune synapses are well-organized and tightly regulated three-dimensional structures composed of different regions called the central, peripheral, and distal supramolecular activation clusters (cSMAC, pSMAC and dSMAC, respectively) [21]. In the context of BiTE molecules and other T-cell-dependent bispecific antibodies, it has been shown that induced immune synapses show hallmarks of maturity such as characteristic LFA-1 ring structures in the pSMAC and exclusion of CD45 from the cSMAC [17, 22].

In this study, we set out to investigate CD33 BiTE molecule-mediated interplay between T cells and target cells. Firstly, we focused on the influence of co-stimulatory and -inhibitory molecules on BiTE molecule-induced formation of immune synapses and their stability, the subsequent T-cell responses, and intracellular downstream signaling in T cells. For this, we established a cell-based model system using the murine pro-B-cell line Ba/F3, which is devoid of human cross-reactive checkpoint molecules, and integrated different sets of co-stimulatory and -inhibitory molecules in addition to the target antigen CD33. Secondly, we translated our findings to primary AML cells for which we modified the expression profile to mimic different clinically relevant phenotypes. Finally, using the immunomodulatory drug (IMiD) lenalidomide, we developed strategies to reverse the impairment of BiTE molecule-mediated T-cell responses that are modulated by the expression profile of AML cells.

## Methods

### Patient and healthy donor material

Peripheral blood mononuclear cells (PBMCs) and bone marrow mononuclear cells (BMMCs) were isolated from peripheral blood (PB) and bone marrow (BM), respectively, using density gradient centrifugation over Histopaque (Sigma-Aldrich, St. Louis, MO, USA). Cell surface expression of CD80, CD86 and PD-L1 was assessed in the Laboratory of Leukemia Diagnostics, University Hospital, LMU Munich, by flow-cytometry (Navios, Beckman Coulter) from > 300 PB and BM samples obtained at the time of first diagnosis or relapse.

### Cell lines

Ba/F3 cells were purchased from the Deutsche Sammlung von Mikroorganismen und Zellkulturen (Leibniz-Institut DSMZ, Braunschweig, Germany) and cultivated under standard conditions in RPMI 1640 supplemented with 10% FBS, 0.5 mg/ml penicillin–streptomycin–glutamine (PSG; Thermo Fisher Scientific, 10,378,016, Waltham, MA, USA), 10 mM HEPES (Carl Roth, HN78.1), and 10% WEHI-conditioned medium. Phoenix-Eco cells were kindly provided by K. Spiekermann (University Hospital, LMU Munich). Phoenix-Eco cells were cultivated in DMEM (Thermo Fisher Scientific, 21,969,035, Waltham, MA, USA) containing 10% FBS and 0.5 mg/ml PSG.

### Primary AML samples

Cryo-preserved patient samples were thawed and cultivated with irradiated MS-5 cells as previously described [9]. Samples were cultivated in *α*-MEM (PAN Biotech, P04-21500, Aidenbach, Germany) supplemented with 12.5% FBS, 12.5% horse serum (Sigma-Aldrich, H1270, St. Louis, MO, USA), and 0.5 mg/ml PSG. The medium was additionally supplemented with 20 ng/ml recombinant human (rhu) granulocyte colony-stimulating factor (Peprotech, 300–23, Hamburg, Germany), rhu interleukin 3 (Peprotech, 200–03, Hamburg, Germany), rhu thrombopoietin (Peprotech, 300–18, Hamburg, Germany), and 57.2 µM 2-mercaptoethanol (Sigma-Aldrich, 63,689, St. Louis, MO, USA).

For some experiments, 25 ng/ml IFN*γ* and 50 ng/ml TNF*α* (both Peprotech, Hamburg, Germany) were added to AML cells after 24 h. After a total pre-culture time of 72 h, T cells were magnetically depleted from the cell suspension (Stemcell Technologies, Vancouver, Canada) and AML cells were used as described in the following sections. Detailed patient characteristics obtained for ex vivo evaluation is summarized in supplementary Table S2.

### DNA constructs and vectors

Human cDNA encoding CD80, CD86 and PD-L1 was purchased from R&D Systems (RDC1086, RDC0693 and RDC1087, respectively) and subcloned into the retroviral expression vector MSCV-neo. Human CD33 cDNA was subcloned into the MSCV-IRES-GFP vector (both kindly gifted by K. Spiekermann).

### Transfection of Phoenix-Eco cells

Phoenix-Eco cells (6.5 × 10^6^) were seeded in a 10 mm Petri dish 1 day prior to transfection. Subcloned plasmid DNA was transiently transfected using calcium phosphate co-precipitation. Medium was replenished after incubation for 18 h, and after another 30 h retroviral supernatant was collected.

### Transduction of Ba/F3 cells

Ba/F3 cells (1.5 ml) were seeded at a density of 5 × 10^5^/ml in six-well plates. Transduction was performed by adding 1.5 ml of retroviral supernatant in presence of polybrene (8 µg/ml). Cells were spun (90 min, 1565 rcf), then cultivated for 72 h, and positive cell fractions were sorted using fluorescence-activated cell sorting (FACS). Quantification of antigen expression was determined using QuantiBRITE PE Beads (BD Biosciences, Franklin Lakes, NJ, USA) according to manufacturer’s instructions. All antibodies used for flow cytometry and FACS staining are listed in supplementary Table S1.

### T-cell-mediated cytotoxicity assay

Redirected cytotoxicity was evaluated in co-cultures of Ba/F3 sublines or primary AML samples and different T-cell subsets in the presence of AMG 330 (0.5–5 ng/ml) or a control construct (c BiTE; specificity against an herbicide. Provided by AMGEN) with an effector to target cell ratio (E:T) of 1:4–1:1. T-cell subsets were either sorted by FACS (CD3^+^, CCR7^+^CD45RA^+^ and CCR7^+^CD45RA^−^) or isolated magnetically and untouched (pan T cells, CD4^+^, CD8^+^) according to the manufacturer’s recommendations (Stemcell Technologies, Vancouver, Canada). In some experiments, T-cells were labeled with CellTrace Far Red (Thermo Fisher Scientific, Waltham, MA, USA) according to the manufacturer’s recommendations. After 3 days, cytotoxicity, T-cell proliferation, granzyme B expression and cytokine secretion were determined using multiparameter flow cytometry (MPFC) (CytoFLEX, Beckman Coulter). Specific lysis was calculated as follows: % specific lysis = 100–[number of viable CD33^+^ target cells (AMG 330 condition)]/[number of viable CD33^+^ target cells (c BiTE condition)] × 100. T-cell proliferation was determined by dilution of membrane dye (% proliferation in AMG 330 condition set to c BiTE condition as baseline) or calculation of relative T-cell numbers on day 3 compared to day 0. Granzyme B expression was assessed by intracellular cytokine staining. IFN*γ*, TNF*α* and IL-2 secretion was measured by analyzing co-culture’s supernatant by cytometric bead array according to the manufacturer’s instructions (BD Biosciences, Franklin Lakes, NJ, USA).

### In vivo mouse studies

Ba/F3 CD33^+^ or Ba/F3 CD33^+^ CD86^+^ sublines (1 × 10^7^) were injected with T cells (1 × 10^7^) intravenously into the tail vain of 8–12-weeks-old female and male NSG mice (own breeding or Janvier Labs, Le Genest-Saint-Isle, France). Mice were treated either with AMG 330 or c BiTE (200 µg/kg) and sacrificed after 24 h. Samples from bone marrow and spleen were collected and analyzed by MPFC. Specific lysis was calculated as follows: % specific lysis = 100–[mean of number of viable CD33^+^ target cells (AMG 330 condition)]/[mean of number of viable CD33^+^ target cells (c BiTE condition)] × 100. All animal experiments were approved by the local regulatory agency (Regierung von Oberbayern).

### Phospho-flow and imaging flow cytometry

Cell conjugates were formed by seeding 0.5–2 × 10^6^ cells/ml (100–200 µl) with an E:T ratio of 1:1. For phospho-flow analysis, cells were spun (30 s, 550 rcf) immediately after adding AMG 330 (5 ng/ml) to the cell suspension. Cells were gently resuspended by flicking the tube, and reactions were stopped with the addition of fixation buffer (BD Biosciences, Franklin Lakes, NJ, USA) after a total of 1, 3, 5, 10 or 20 min. Cells were fixed for 15 min at 37 °C, spun (5 min, 550 rcf) and permeabilized with methanol (BD Biosciences, Franklin Lakes, NJ, USA) for 30 min on ice. Afterwards, cell conjugates were stained against CD45, pERK1/2, pAkt, and pZAP70 and analyzed by flow cytometry. For imaging flow cytometry (Amnis ImageStream Mk II, Luminex) cell conjugation was stopped after 1 h. Twenty minutes before fixation, LFA-1 staining antibody was added to the reaction. Fixed cells were spun (5 min, 550 rcf), stained for CD45, and Hoechst 33,342 (Thermo Fisher Scientific, Waltham, MA, USA) was added. In the case of primary AML samples, target cells were labeled with CellTrace CFSE (Thermo Fisher Scientific, Waltham, MA, USA) before conjugate formation.

### Spinning disc confocal microscopy

To study TCR triggering, we used a reconstituted T-cell system previously described by James et al. [23]. Briefly, for these experiments, HEK-T cells are non-immune HEK293T cells expressing the TCR complex and CD45 genetically fused to eGFP. HEK-T cells (1 × 10^5^ cells) were conjugated for 20 min with CD33-transduced Raji B cells (2 × 10^5^ cells) in the presence of either AMG 330 (1.25 µg/ml) or a c BiTE construct, which had been fluorescently labeled with Alexa Fluor 647. The Raji B cells expressed mTagBFP for identification, and the transduced CD33 was tagged with mScarlet so its clustering could be directly visualized. Spinning disk confocal microscopy (Andor) was used to image the cell conjugates at 37 °C. All images were analyzed, and all presented images were manipulated in an equivalent manner using ImageJ. The level of protein clustering and segregation of denoted proteins was measured by manually defining the intensity of fluorescently labeled proteins in the plasma membrane within the cell–cell interface.

### Metabolic analysis

CD33^+^ cells were depleted from T cell–Ba/F3 cell co-cultures using a Human CD33 Positive Selection Kit (Stemcell Technologies, Vancouver, Canada) according to the manufacturer’s instructions, and the remaining T cells (2.5 × 10^5^ T cells/well) were plated on a poly-D-lysine-coated 96-well Seahorse utility plate (Agilent, Santa Clara, CA, USA). Metabolic functions were analyzed in standard mitochondrial and glycolysis stress tests on a Seahorse XFe96 Analyzer using appropriate kits (Agilent, Santa Clara, CA, USA, respectively). Final concentration for the mitochondrial stress test for oligomycin, FCCP and rotenone/antimycin A were 1 µM, each. For the glycolysis stress test a final concentration of 10 mM glucose, 1 µM oligomycin and 50 mM 2-DG were used. Positive control was performed with pan T cells activated for 72 h with Human T-Activation CD3/CD28 Dynabeads (Thermo Fisher Scientific, Waltham, MA, USA) at a 1:2 beads:cell ratio. Untreated pan T cells served as a negative control.

### Data analysis

Flow cytometry data were analyzed using FlowJo software (BD Biosciences, version 10) and imaging flow cytometry data were analyzed with IDEAS 3.0 (Amnis). GraphPad Prism was used to perform statistical evaluations (GraphPad, version 9.3). ImageJ was used to analyze spinning disc confocal microscopy images. TIMER2.0 was used to perform analysis between gene expression and clinical outcome [24].

## Results

### T-cell co-signaling ligands are expressed on leukemic cells

**Fig. 1:**
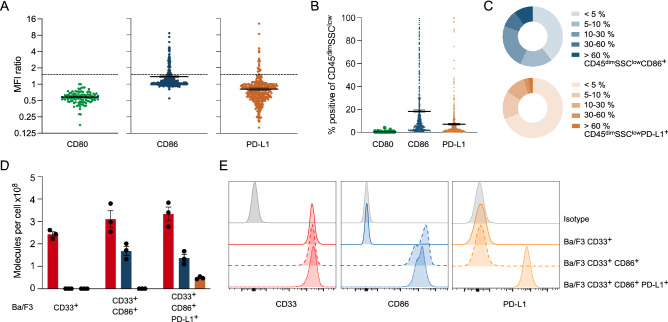
T-cell co-signaling receptors are expressed on leukemic cells and transduced Ba/F3 sublines. **A** MFI ratio of cell surface expression of CD80 (green), CD86 (blue) and PD-L1 (orange) in CD45^dim^SSC^low^ primary AML samples (*n* = 107–377). MFI ratios ≥ 1.5 (dashed lines) indicate positivity. **B** Percentage of CD80-, CD86- and PD-L1-positive cells and **C** distribution of CD86 and PD-L1 expression intensities within primary AML samples (*n* = 432–521). **D** Antigen count of CD33 (red), CD86 (blue) and PD-L1 (orange) on transduced Ba/F3 sublines (*n* = 3) with **E** representative histograms

Cell surface expression of the T-cell co-signaling ligands CD80, CD86 and PD-L1 was assessed on leukemic bulk cells (CD45^dim^SSC^low^) from peripheral blood (PB) and bone marrow (BM) samples obtained at the time of first diagnosis or relapse. All analyzed samples were negative for cell surface expression of CD80 (Fig. 1A). We observed CD86 expression in 61 of 333 (18.32% with an MFI ratio > 1.5; Fig. 1A) and PD-L1 expression in 21 of 377 (5.57% with an MFI ratio > 1.5; Fig. 1A) primary AML samples. Patient samples showed a variability in their expression levels, and we report these subdivided into the five groups defined by Tamura et al. [25]: < 5%, 5–10%, 10–30%, 30–60% and > 60% (Fig. 1B, C).

Based on these findings, we decided to investigate the roles of CD86 and PD-L1 in a cell-based model system. For this, we generated a stable expression system by transducing the murine pro-B cell line Ba/F3 with human (hu) CD33 (2.4–3.1 × 10^8^ molecules/cell) ± huCD86 (1.4–1.7 × 10^8^ molecules/cell) ± huPD-L1 (4.8 × 10^7^ molecules/cell) (Fig. 1D, E). Expression levels of CD86 and PD-L1 are higher on Ba/F3 sublines compared to primary AML samples. However, the CD86/PD-L1 ratio of mean expression (~ 1.7) were comparable.

### AMG 330 induces TCR triggering characterized by CD45 exclusion from and CD33 clustering within the synapse

**Fig. 2:**
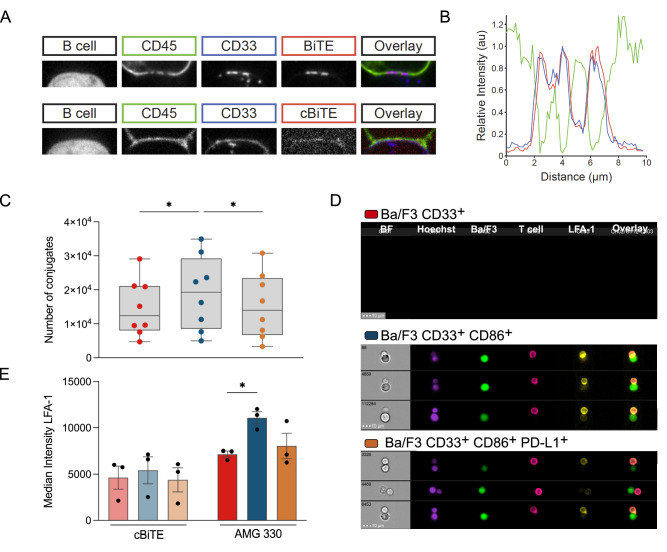
AMG 330 induces TCR triggering characterized by CD45 exclusion from and CD33 clustering within the synapse. **A** Representative spinning disc confocal microscope images of AMG 330 (BiTE^®^ molecule) and c BiTE molecule-mediated conjugates formed of a CD33-transduced Raji B cell and a reconstituted HEK-T cell. **B** Line profiles of CD45 (green), CD33 (blue), and AMG 330 (red) intensities across a conjugate interface equivalent to that shown in a representative image in panel A. **C** Total number of AMG 330-induced T-cell–CD33^+^ CD86^±^ PD-L1^±^ Ba/F3 cell conjugates after 20 min, assessed by flow cytometry. **D** Representative imaging flow cytometric analysis of AMG 330-induced T-cell–CD33^+^ CD86^±^ PD-L1^±^ Ba/F3 cell conjugation: brightfield (BF, gray), Hoechst staining (purple), Ba/F3 cell (GFP^+^; green), T cell (CD45^+^; magenta), LFA-1 (yellow), and overlay of Ba/F3, T-cell and LFA-1 channels. **E** Median intensity of LFA-1 accumulation at the interface of AMG 330-and c BiTE molecule-induced T-cell–CD33^+^ CD86^±^ PD-L1^±^ Ba/F3 cell conjugates. Statistical analysis: One-way ANOVA with Dunnett's multiple comparisons test; ns *p* > 0.05, *p ≤ 0.05

Using a reconstituted T-cell system previously described by James et al. [23], we characterized the immune synapse that forms upon AMG 330-induced TCR triggering. We observed exclusion of CD45 from the immune synapse and simultaneous CD33 clustering within the Raji B–HEK T-cell interface in the presence of AMG 330, but not in the presence of c BiTE (Fig. 2A). Quantification of the relative fluorescence signal intensities of CD45, CD33 and AMG 330 within the cell–cell interface revealed that CD33 clustering and AMG 330 binding were co-localized. In contrast, CD45 exclusion was spatially distinct and clearly anti-correlated (Fig. 2B). Next, we investigated AMG 330-mediated conjugation and stability of synapse formation after 20 and 60 min, respectively, with different Ba/F3 sublines. The total number of conjugates formed with CD33^+^ CD86^+^ Ba/F3 subline was approximately 1.3-fold higher than with those expressing either no co-stimulatory antigen or additional PD-L1 in the presence of AMG 330 (Fig. 4A). Furthermore, expression of CD86 without PD-L1 on Ba/F3 CD33^+^ subline resulted in a remarkable increase of LFA-1 accumulation at the cell–cell interface, determined by imaging flow cytometry (for gating strategy see supplementary Fig. S1). In contrast, LFA-1 expression intensity within Ba/F3 CD33^+^ PD-L1^±^–T cell conjugates was comparable to the control with c BiTE (Fig. 2C, D). Collectively, our results demonstrate that expression of the co-stimulatory molecule CD86 stabilizes immune synapses, as seen by the accumulation of LFA-1 at the cell–cell interface, whereas concomitant expression of PD-L1 had a negative impact on the formation of conjugates and synapses.

### Checkpoint molecule expression on target cells modulates AMG 330-mediated cytotoxicity and T-cell function

**Fig. 3:**
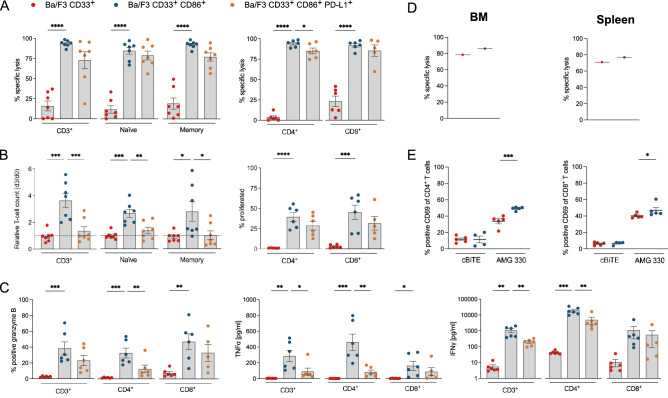
Checkpoint molecule expression on target cells modulates AMG 330-mediated cytotoxicity and T-cell function. **A** AMG 330-mediated cytolytic capacity, **B** proliferation, **C** granzyme B expression and cytokine secretion of HD T-cell subsets after co-culture with CD33^+^ CD86^±^ PD-L1^±^ Ba/F3 sublines for 72 h. **D** AMG 330-mediated cytolytic capacity in BM and spleen and **E** CD69 expression of HD T cells (from BM) against CD33^+^ CD86^±^ sublines in vivo. AMG 330 concentration = 0.5–5 ng/ml (in vitro) or 200 µg/kg (in vivo); E:T ratio = 1:1; *n* = 5–6; Error bars represent mean ± SEM; Statistical analysis: One-way ANOVA or Mixed-effects analysis with Dunnett's multiple comparisons test; ns *p* > 0.05, *p ≤ 0.05, **p ≤ 0.01, ***p ≤ 0.001, **** *p* ≤ 0.0001

In co-culture assays with different Ba/F3 sublines and healthy donor (HD) T cells the potential for CD86 and PD-L1 to modulate T-cell cytotoxicity and AMG 330-redirected T-cell function was evaluated. We observed that lysis of CD33 single-positive Ba/F3 cells upon addition of AMG 330 and HD T cells was < 25% after 3 days. Cytotoxicity was significantly improved by co-expression of CD86 (Fig. 3A). Higher cytotoxicity against Ba/F3 CD33^+^ CD86^+^ was accompanied by significant increases in T-cell proliferation, expression of granzyme B, and secretion of IFN*γ* and TNF*α* (Fig. 3B, C). HD T cells did not induce lysis against Ba/F3 cells due to xenogeneic MHC recognition (Fig. S2C). Furthermore, we were able to translate our findings into an in vivo model system. Specifically, we observed higher clearance of the Ba/F3 CD33^+^ CD86^+^ subline compared to the Ba/F3 CD33^+^ subline in the BM and spleen in an orthotopic mouse model (Fig. 3D). In line with these observations, T cells from Ba/F3 CD33^+^ CD86^+^ mice also showed higher CD69 expression levels (Fig. 3E). The influence of co-inhibition was investigated in co-cultures with the CD33^+^ CD86^+^ PD-L1^+^ Ba/F3 subline. Although this resulted in an only marginal decrease of AMG 330-mediated cytotoxicity, T-cell proliferation and function were markedly reduced (Fig. 3A–C). These findings could be observed in bulk T cells and in CD4^+^, CD8^+^, naïve and memory T-cell subpopulations used for co-culture. Our findings underline the importance of co-stimulatory molecules in AMG 330-mediated cytotoxicity against CD33-expressing target cells.

### AMG 330-induced downstream signaling in T cells is strongly enhanced by CD86 expression on target cells

**Fig. 4:**
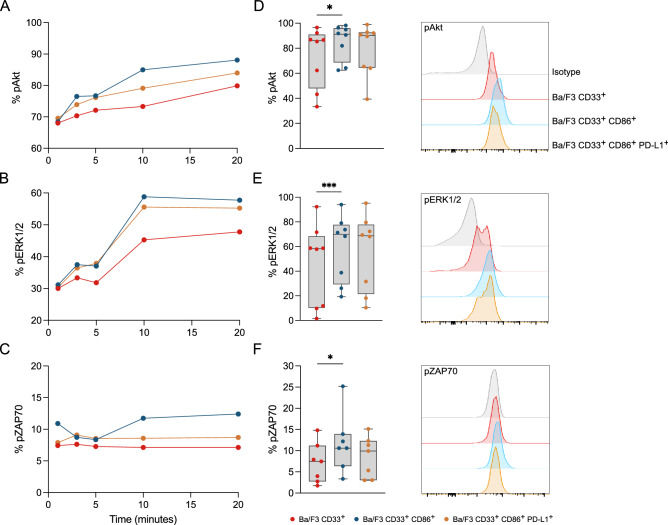
AMG 330-induced downstream signaling in T cells is strongly enhanced by CD86 expression on target cells. Kinetics of phosphorylation (% positive) of **A** Akt, **B** ERK1/2, and **C** ZAP70 in T cells after 1, 3, 5, 10, and 20 min of AMG 330-mediated engagement with different Ba/F3 cell constructs. Percentage of phosphorylated **D** Akt, **E** ERK1/2, and **F** ZAP70 in T cells after 10 min of engagement. Overlays of representative flow cytometry histograms after 10 min of engagement are shown. AMG 330 concentration = 5 ng/ml; E:T ratio = 1:1; *n* = 7–8; Error bars represent mean ± SEM; Statistical analysis: One-way ANOVA or Mixed-effects analysis with Dunnett's multiple comparisons test; ns *p* > 0.05, *p ≤ 0.05, **p ≤ 0.01, ***p ≤ 0.001

In a next step, we aimed to characterize downstream signaling in T cells of various kinases that are involved in the regulation of TCR phosphorylation and subsequent T-cell proliferation and survival. Therefore, we measured the phosphorylation of Akt, ERK1/2 and ZAP70 after engagement between HD T cells and different Ba/F3 subline in the presence of AMG 330. The percentage of kinase phosphorylation after 1, 3, 5, 10, and 20 min was determined relative to unstimulated T cells as a background control. We observed time-dependent phosphorylation of Akt, ERK1/2 and ZAP70 within the T cells regardless of which Ba/F3 subline was engaging (Fig. 4A–C). The extent of phosphorylation varied between the T-cell HDs and also depended on the Ba/F3 subline, according to the trend Ba/F3 CD33^+^ CD86^+^  >  > Ba/F3 CD33^+^ CD86^+^ PD-L1^+^  > Ba/F3 CD33^+^. This observation was most pronounced after 10 min of conjugate formation (Fig. 4D–F).

### PD-L1 expression on target cells alters metabolic reprogramming of T cells

**Fig. 5:**
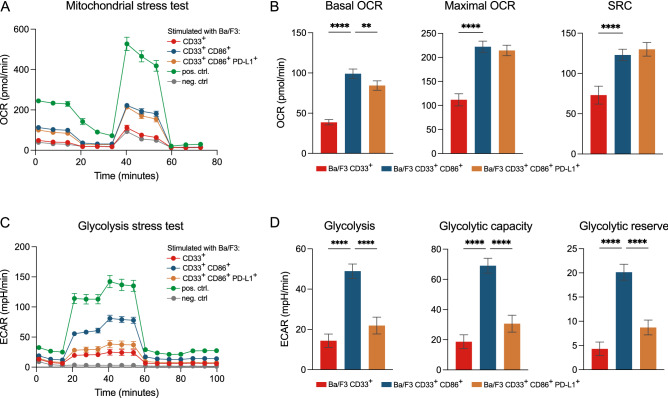
PD-L1 expression on target cells alters metabolic reprogramming of T-cells. **A** OCR kinetics of T cells after 72 h of co-culture with different Ba/F3 sublines in the presence of AMG 330, after 72 h of stimulation with CD3/CD28 beads (pos. ctrl) or left unstimulated for 72 h (neg. ctrl); **B** corresponding bar graphs showing basal OCR, maximal OCR, and SRC in a mitochondrial stress test. **C** Kinetics of ECAR of T cells after 72 h of co-culture with different Ba/F3 sublines in the presence of AMG 330, after 72 h of stimulation with CD3/CD28 beads (pos. ctrl) or left unstimulated for 72 h (neg. ctrl); **D **corresponding bar graphs showing glycolysis, glycolytic capacity, and glycolytic reserve in a glycolysis stress test. AMG 330 concentration = 0.5 ng/ml; E:T ratio = 1:1; *n* = 5; Error bars represent mean ± SEM; Statistical analysis: Mixed-effects analysis with Dunnett's multiple comparisons test; ns *p* > 0.05, *p ≤ 0.05, **p ≤ 0.01, ***p ≤ 0.001, ****p ≤ 0.0001

Activation of T cells is accompanied by a metabolic reprogramming and is further modulated by the engagement of ligands expressed on APCs [26]. T cells activated by AMG 330 in co-culture with the CD33^+^ Ba/F3 subline, devoid of positive co-stimulatory molecules, showed very low metabolic activity with low oxygen consumption rates (OCRs; Fig. 5A) and extracellular acidification rates (ECARs; Fig. 5C). By contrast, T cells activated by AMG 330 in co-culture with the CD33^+^ CD86^+^ Ba/F3 subline, showed a significant increase of both glycolysis and oxidative phosphorylation, indicating high metabolic activity (Fig. 5A–D). T cells stimulated with the CD33^+^ CD86^+^ PD-L1^+^ Ba/F3 subline showed an altered metabolic phenotype, preferring oxidative phosphorylation over glycolysis as the main pathway for energy production. In accordance with a comparable approach, PD-L1 stimulation led to an even higher spare respiratory capacity (SRC) in T cells than CD86 stimulation alone (Fig. 5B) [26]. These data underline that co-stimulation is a prerequisite for achieving a fully functional BiTE molecule-mediated T-cell response.

### IFNγ and TNFα modulate the profile of checkpoint molecule expression of primary AML cells

**Fig. 6:**
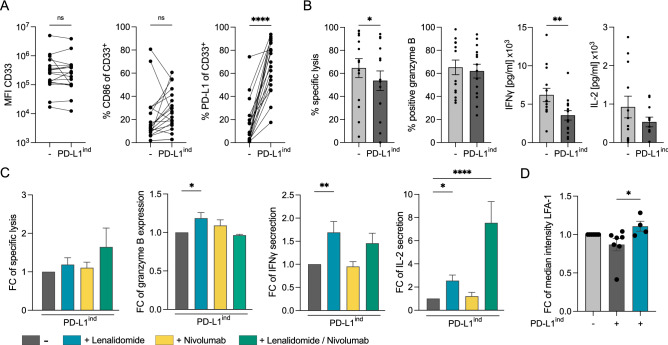
Lenalidomide reverses negative impact of IFN*γ* and TNF*α* on AMG 330-mediated T-cell function and synapse formation in co-cultures with primary AML samples. **A** Expression of CD33 (MFI), CD86 (% CD33^+^), and PD-L1 (% CD33^+^) on primary AML samples after pretreatment ± IFN*γ* and TNF*α* (PD-L1^ind^) for 48 h. **B** AMG 330-mediated cytolytic capacity, granzyme B expression, IFN*γ* and IL-2 secretion of HD T cells against non-pretreated and PD-L1^ind^ primary AML samples. **C** Fold change of AMG 330-mediated cytolytic capacity, granzyme B expression, IFN*γ* and IL-2 secretion of HD T cells against PD-L1^ind^ primary AML samples ± lenalidomide or/and nivolumab after 72 h of co-culture. **D** Fold change of median intensity of LFA-1 accumulation at the interface of AMG 330-induced T-cell–primary AML cell conjugates. Primary AML samples were pretreated ± IFN*γ* and TNF*α* for 48 h and HD T cells were pretreated ± lenalidomide for 24 h; AMG 330 concentration = 5 ng/ml; Lenalidomide = 10 µM; Nivolumab = 10 µg/ml; E:T ratio = 1:4 (panels B and C) or 1:1 (panel D); *n* = 4–14; Error bars represent mean ± SEM; Statistical analysis: Paired t-test (panel A and B) and One-way ANOVA with Dunnett's multiple comparisons test (Panels C and D); ns *p* > 0.05, *p ≤ 0.05, **p ≤ 0.01, ***p ≤ 0.001, ****p ≤ 0.0001

Having found that T-cell co-signaling ligands on target cells influence AMG 330-mediated T-cell function in our cell-based model system, we next aimed to translate our findings into primary patient material. To increase PD-L1 levels in primary AML samples, we pre-cultivated primary AML samples with IFN*γ* and TNF*α* to induce PD-L1 expression (PD-L1^ind^). After 48 h we observed a significant upregulation of PD-L1, whereas expression of CD33 and CD86 was not significantly affected by cytokine treatment (Fig. 6A). This led to significantly reduced AMG 330-mediated cytotoxicity and granzyme B expression, together with a decrease in IFN*γ* and IL-2 secretion (Fig. 6B). Importantly, these observations were accompanied by reduced stability of the immune synapse, as indicated by lower levels of LFA-1 expression within AMG 330-induced cell–cell conjugates after IFNγ and TNF*α* pre-treatment (Fig. 6D).

### Lenalidomide reversed the negative impact of IFNγ and TNFα on AMG 330-mediated T-cell function and synapse formation in co-cultures with primary AML samples

Pre-treatment of primary AML samples with IFN*γ* and TNF*α* led to PD-L1 upregulation and decreased AMG 330-mediated stability of immune synapses. Accordingly, we observed that the AMG 330-mediated function of T cells was diminished in co-cultures with pre-treated AML samples. In a next step, we aimed to reverse this functional impairment due to pro-inflammatory cytokines by using the IMiD lenalidomide (10 µM). Indeed, we could show that T-cell responses and the stability of immune synapses were significantly improved by adding lenalidomide to co-cultures. Interestingly, PD-1 blockade with nivolumab (10 µg/ml) could not abrogate the negative impact of the inflammatory stimuli to the same extent as lenalidomide. However, the combination of PD-1 blockade and lenalidomide mediated the highest specific lysis of primary AML cells and significantly enhanced IL-2 secretion (Fig. 6B–D; Fig. S3). Of note, expression levels of PD-L1 and CD86 on primary AML cells and PD-1 on T-cells remained unchanged through the exposure to lenalidomide (Fig. S3). Our results support the notion that lenalidomide can reverse the negative impact of IFN*γ* and TNF*α* on AMG 330-mediated T-cell responses by improving immune synapse formation and circumventing inhibitory signals on T cells by boosting their effector function, especially in combination with PD-1 blockade.

## Discussion

Since the approval of blinatumomab—the first bispecific T-cell-redirecting antibody molecule—other bispecific constructs of different formats have been evaluated in several clinical trials. Antibodies including mosunetuzumab have entered clinical practice in the setting of r/r B-cell lymphoma. Tebentafusp, a bispecific fusion protein, was granted approval for treating uveal melanoma, and other bispecific T-cell-redirecting agents are expected to follow in hematological and solid malignancies [6–8, 27–30].

Despite these impressive developments, a significant number of patients do not respond to this type of therapy, or they eventually relapse. It is well known that the efficacy of bispecific T-cell-redirecting antibodies crucially depends on T-cell activation and proliferation, although the parameters determining these activities are poorly understood [31]. Therefore, it is of critical importance to better understand the contributors to an effective T-cell response for improving treatment strategies involving T-cell-redirecting antibodies.

Here, we present a unique model cellular platform based on the murine Ba/F3 cell line aimed at deepening our insight into the mechanistic aspects of T-cell activation mediated by CD33 BiTE molecules. This model system is free from interfering endogenous stimulatory or inhibitory checkpoint molecules (Fig. S4) and provides an ideal setup for understanding how selected surface molecules are involved in the formation of synapses and subsequent T-cell activation and downstream signaling.

Specifically, our cell-based platform integrates different T-cell co-signaling ligands in combination with an AML-associated target antigen to mimic the expression profile of primary AML samples. To achieve this, we screened the surface expression of > 300 primary AML samples for the T-cell co-signaling ligands CD80, CD86 and PD-L1 by MPFC. Furthermore, we correlated gene expression to overall survival (OS) for AML patients. PD-L1^high^ patients have worse OS than PD-L1^low^ patients (Fig. S4).

Based on this analysis, we integrated CD86 and PD-L1 into our model system. Notably, whereas the expression of CD80 and CD86 has been reported at varying levels, [25, 32, 33] our analysis showed a smaller proportion of patients with CD86 expression and, in line with the study by Maeda et al. [34], a lack of CD80 expression in primary AML samples.

Next, by using a reconstituted T-cell system, we demonstrated that AMG 330 induces TCR triggering, showing hallmarks of the formation of mature immune synapses, such as the exclusion of CD45 and enrichment of CD33 at the cell–cell interface [22, 23]. Another characteristic of maturation and stability of an immune synapse is the enrichment of LFA-1 within the cell–cell surface area of engagement [35, 36]. Importantly, human LFA-1 binds to murine ICAM-1 [37] enabling a cross-species assay setup to study human antigen-specific BiTE molecules and their impact on synapse stability. Indeed, LFA-1 expression intensities were highest in the Ba/F3 CD33^+^ CD86^+^ cell–T cell conjugates. As a consequence, target cell lines expressing CD86 elicited significantly higher BiTE molecule-mediated cytotoxicity, T-cell proliferation, granzyme B expression and cytokine secretion than those expressing only the target antigen or in combination with PD-L1. Surprisingly, CD4^+^ T cells exhibited similar levels of cytotoxicity and T-cell proliferation in comparison to CD8^+^ T cells against the Ba/F3 CD33^+^ CD86^+^ subline. In line with these observations, Melenhorst et. al. [38] reported on a dominance of CD4^+^ CAR T-cells subsets in CLL patients with long lasting remission. Also, previous reports in antiviral immunity demonstrated cytolytic function within the CD4 T-cell compartment [39, 40].

It is well known that T cells undergo a reprogramming of different metabolic pathways depending on the stimulus they encounter during their activation, which shapes their long-term survival and persistence [41–43]. We provide evidence that the absence of co-stimulation leads to T cells entering a metabolically inactive state in the presence of BiTE molecules, whereas stimulation by CD86 promotes a switch to glycolysis to meet the nutritional demand of highly functional effector T cells. Consistent with a reduced effector function, T cells stimulated additionally with PD-L1 generate less energy by glycolysis, but remarkedly increased their oxidative phosphorylation with high SRC, as reported for physiological APC–T-cell interactions mediated by PD-1–PD-L1 ligation [26].

Our cell-based model system is devoid of interfering molecules, enabling the impact of CD86 and PD-L1 on BiTE molecule-mediated downstream signaling in T cells to be isolated. We tracked the phosphorylation of ZAP70, Akt and ERK1/2, the main contributors to the activation cascade, and demonstrated that T-cell co-signaling ligands predetermine CD33 BiTE molecule-mediated T-cell function. These observations underline the essential nature of signal 2 (co-stimulation) in CD33 BiTE molecule-mediated T-cell responses and suggest that the expression profiles of patient-derived samples contribute to the efficacy of bispecific T-cell-redirecting molecules.

Finally, we aimed to validate our findings from the cell-based model system in primary AML samples. In response to redirected T-cell activation and linked pro-inflammatory cytokine secretion, the expression of PD-L1, among others, is a mechanism of the adaptive immune response in various malignancies. We conditioned primary AML cells with IFN*γ* and TNF*α*, which resulted in the upregulation of PD-L1 accompanied by impaired T-cell-redirected cytotoxicity and immune synapse formation by the BiTE molecule AMG 330.

Our observations are consistent with other preclinical studies attempting to overcome the detrimental impact PD-L1 has on T-cell responses mediated by bispecific antibodies [18, 44, 45]. The concept of combining bispecific T-cell-redirecting antibodies with checkpoint inhibition in AML is currently being evaluated in early clinical trials, however, only limited data exists to assess its effectiveness. Therefore, we sought to implement a novel combinatorial approach for overcoming the loss of functionality of T-cells acquired during treatment with redirecting bispecific antibodies. Based on our findings, we hypothesized that we could improve BiTE molecule-mediated T-cell responses by modulating immune synapse formation.

In fact, by introducing lenalidomide into our *in vitro* model system with primary AML cells, we were able to significantly enhance CD33 BiTE molecule-mediated T-cell cytotoxicity and cytokine secretion. In accordance with reports on T-cell defects in chronic lymphocytic leukemia (CLL) due to impaired LFA-1 activation and motility, we observed an improvement in the stability of immune synapses through the addition of lenalidomide [46, 47]. Together, this supports the concept of combining bispecific T-cell-redirecting molecules with an IMiD to increase their efficacy.

Our study provides a unique and reliable cell-based model system with which to unravel the molecular mechanisms as redirected by CD33 BiTE molecules. This system is highly suitable for investigating and deciphering their mode of action as it can be modulated to express nearly any desired surface antigen in different combinations [48, 49]. Thereby, we were able to demonstrate the impact of co-stimulatory and co-inhibitory molecules on synapse formation and subsequent T-cell responses. Furthermore, we translated our findings from the cell-based model to primary patient material, underlining the notion that the expression profile of the target cells modulates the efficacy of T-cell-redirecting antibodies. By combining the antibody treatment with an IMiD, we were able to enhance impaired T-cell responses and observed an improvement in immune synapse formation, supporting the potential use of combinational strategies in future clinical trials.

### Supplementary Information

Below is the link to the electronic supplementary material.

Supplementary file1 (DOCX 1440 KB)

## Data Availability

The datasets analyzed during the current study are available from the corresponding author on reasonable request.

## Abbreviations

AML: Acute myeloid leukemia
APC: Antigen presenting cell
BCP-ALL: B-cell precursor acute lymphoblastic leukemia
BM: Bone marrow
BMMC: Bone marrow mononuclear cell
(c)BiTE: (Control) bispecific T-cell engager
cSMAC/dSMAC/pSMAC: Central/distal/peripheral supramolecular activation cluster
ECAR: Extracellular acidification rate
FACS: Fluorescence-activated cell sorting
HD: Healthy donor
IMiD: Immunomodulatory drug
MFI: Median fluorescence intensity
MRD^+^: Minimal residual disease-positive
OCR: Oxygen consumption rate
OS: Overall survival
PB: Peripheral blood
PBMC: Peripheral blood mononuclear cell
PSG: Penicillin–streptomycin–glutamine
r/r: Relapsed/refractory
SRC: Spare respiratory capacity
TCR: T-cell receptor
