# Correction to: CD33 BiTE^®^ molecule-mediated immune synapse formation and subsequent T-cell activation is determined by the expression profile of activating and inhibitory checkpoint molecules on AML cells

**DOI:** 10.1007/s00262-023-03457-9

**Published:** 2023-05-08

**Authors:** Anetta Marcinek, Bettina Brauchle, Lisa Rohrbacher, Gerulf Hänel, Nora Philipp, Florian Märkl, Thaddäus Strzalkowski, Sonja M. Lacher, Dragica Udiljak, Karsten Spiekermann, Sebastian Theurich, Sebastian Kobold, Roman Kischel, John R. James, Veit L. Bücklein, Marion Subklewe

**Affiliations:** 1 Department of Medicine III, University Hospital, LMU Munich, Munich, Germany; 2 Laboratory for Translational Cancer Immunology, LMU Gene Center, Munich, Germany; 3 German Cancer Consortium (DKTK) and German Cancer Research Center (DKFZ), Heidelberg, Germany; 4 Experimental Leukemia and Lymphoma Research (ELLF), Department of Medicine III, University Hospital, LMU Munich, Munich, Germany; 5 Cancer-and Immunometabolism Research Group, LMU Gene Center, Munich, Germany; 6 Division of Clinical Pharmacology, Department of Medicine IV; Member of the German Center for Lung Research (DZL), University Hospital, LMU, Munich, Germany; 7 AMGEN Research Munich GmbH, Munich, Germany; 8 AMGEN Inc., Thousand Oaks, CA USA; 9 Division of Biomedical Sciences, Warwick Medical School, University of Warwick, Coventry, UK

**Correction to: Cancer Immunology, Immunotherapy** 10.1007/s00262-023-03439-x

The original version of this article unfortunately contained a mistake. Figure 2D is not displayed correctly. In the “BaF3 CD33+” line no cell images are displayed and it seem that something went wrong with the layers of the figure.

**Fig. 2:**
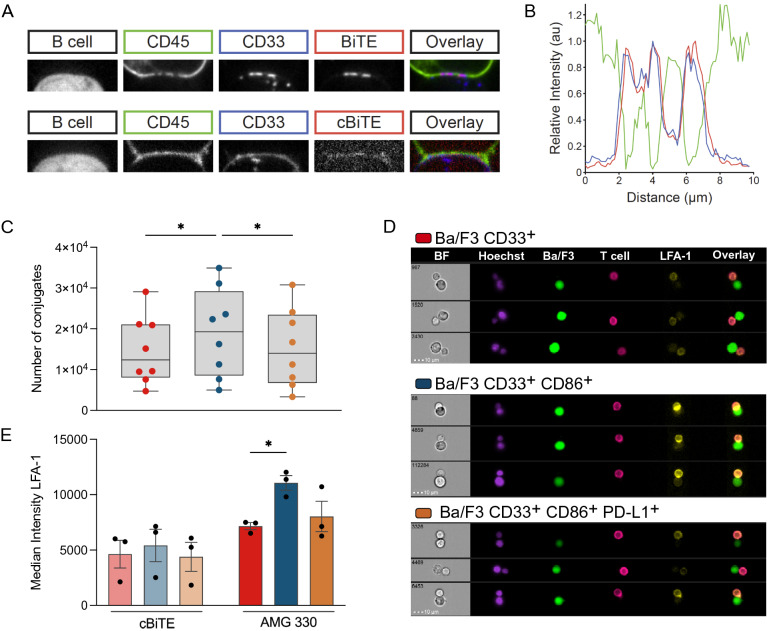
AMG 330 induces TCR triggering characterized by CD45 exclusion from and CD33 clustering within the synapse. **A** Representative spinning disc confocal microscope images of AMG 330 (BiTE^®^ molecule) and c BiTE molecule-mediated conjugates formed of a CD33-transduced Raji B cell and a reconstituted HEK-T cell. **B** Line profiles of CD45 (green), CD33 (blue), and AMG 330 (red) intensities across a conjugate interface equivalent to that shown in a representative image in panel A. **C** Total number of AMG 330-induced T-cell–CD33^+^ CD86^±^ PD-L1^±^ Ba/F3 cell conjugates after 20 min, assessed by flow cytometry. **D** Representative imaging flow cytometric analysis of AMG 330-induced T-cell–CD33^+^ CD86^±^ PD-L1^±^ Ba/F3 cell conjugation: brightfield (BF, gray), Hoechst staining (purple), Ba/F3 cell (GFP^+^; green), T cell (CD45^+^; magenta), LFA-1 (yellow), and overlay of Ba/F3, T-cell and LFA-1 channels. **E** Median intensity of LFA-1 accumulation at the interface of AMG 330-and c BiTE molecule-induced T-cell–CD33^+^ CD86^±^ PD-L1^±^ Ba/F3 cell conjugates. Statistical analysis: One-way ANOVA with Dunnett's multiple comparisons test; ns *p* > 0.05, *p ≤ 0.05

The corrected Fig. 2 is given in the next page.

