# Supplementary material for: CD33 BiTE® molecule-mediated immune synapse formation and subsequent T-cell activation is determined by the expression profile of activating and inhibitory checkpoint molecules on AML cells
Source: Cancer Immunol Immunother. 2023 Apr 11;72(7):2499–512. doi: 10.1007/s00262-023-03439-x (PMC10264534; doi:10.1007/s00262-023-03439-x)
Supplement: Supplementary file 1 — Supplementary file1 (DOCX 1440 KB) [file 262_2023_3439_MOESM1_ESM.docx]

Supplementary Information

### Supplementary Figure S1


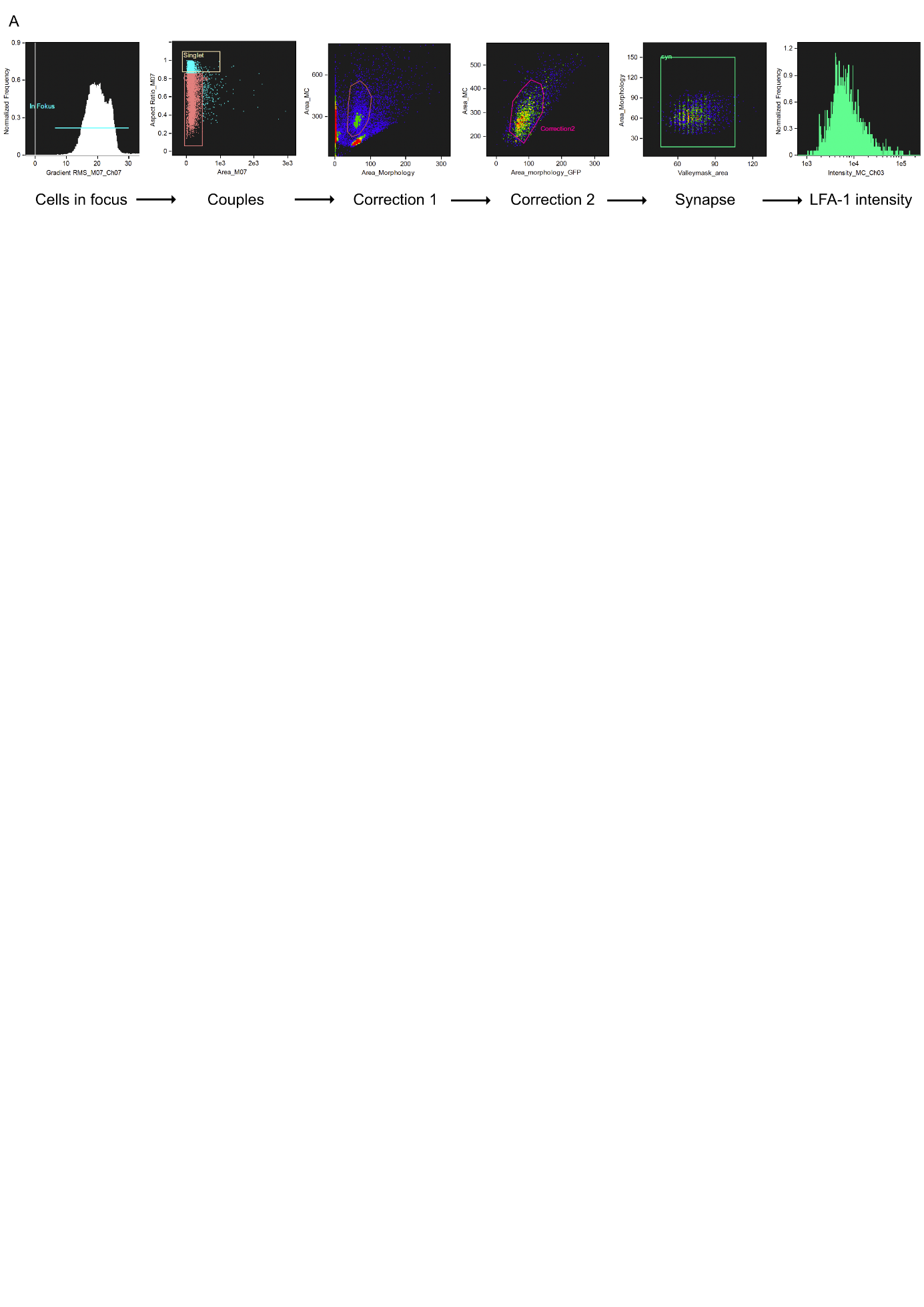


**Figure S1: Gating strategy to quantify LFA-1 intensity in cell-cell conjugates by imaging flow cytometry (A)** Gating of cells in focus by ‘Gradient RMS_M07_Ch07’ followed by discriminating **(B)** single cells from couples by ‘Area_M07’ against ‘Aspect Ratio_M07’. **(C)** Non-T cell–T-cell couples (correction 1) were discriminated by ‘Area_Morphology’ (defined by mask: morphology (M12, Ch12)) against ‘Area_MC’. **(D)** A second correction (correction 2) was applied by gating non-target cell–target cell couples by ‘Area_Morphology_GFP’ (defined by mask: morphology(M02,Ch02)) against ‘Area_MC’. **(E)** Gating of cell couples that formed an immune synapse by ‘Valleymask_area’ (defined by mask: valley (M07, Ch07,3)) against ‘Area_Morphology’. **(F)** Histogram to define median of LFA-1 intensity by ‘Intensity_MC_CH03’. Channels used for analysis: Ch02 (GFP or CFSE), Ch03 (LFA-1 in PE), Ch07 (Hoechst), Ch12 (CD45 in APC/Cy7).

### Supplementary Figure S2

**Figure S2: (A)** Representative flow cytometry dot plots of co-cultures of HD T cells with CD33^+^ CD86^±^ Ba/F3 cells in the presence of AMG 330/ cBiTE molecule after 72 h and overlay of representative flow cytometry histograms of AMG 330 and c BiTE molecule-mediated granzyme B expression and T-cell proliferation after co-culture with different Ba/F3 cells for 72 h. **(B)** Total number of AMG 330-induced T-cell–CD33^+^ CD86^±^ PD-L1^±^ Ba/F3 cell conjugates ± lenalidomide after 20 min assessed by flow cytometry. **(C)** Fold change of viable cell count of Ba/F3 sublines after co-culture with HD T cells for 72 h days compared to Ba/F3 sublines cultivated without HD T cells. AMG 330 concentration=5 ng/ml; E:T ratio=1:1; *n*=3–12; Error bars represent mean ± SEM; Statistical analysis: One-way ANOVA with Dunnett's multiple comparisons test; ns *p* > 0.05, **p* ≤ 0.05, ***p* ≤ 0.01.

**Supplementary Figure S3**

**Figure S3: (A)** Expression of PD-L1 on primary AML samples after pre-treatment ± IFNγ, TNFα and Lenalidomide for 48 h. **(B)** Expression of CD86 and PD-L1 on primary AML samples that were either pre-treated with IFNγ and TNFα (PD-L1^ind^) or not after 3 days of culture ± lenalidomide and PD-1 expression on T-cells after co-culture with primary AML samples in presence of AMG 330 ± lenalidomide.**(C)** Fold change of AMG 330-mediated cytolytic capacity, granzyme B expression, IFNγ and IL-2 secretion of HD T cells against non-pretreated primary AML samples ± lenalidomide or/and nivolumab after 72 h of co-culture; AMG 330 concentration=5 ng/ml; Lenalidomide=10 µM; Nivolumab=10 µg/ml; E:T ratio=1:4; *n*=3–8; Error bars represent mean ± SEM; Statistical analysis: One-way ANOVA with Dunnett's multiple comparisons test; ns *p* > 0.05, **p* ≤ 0.05, ***p* ≤ 0.01.

**Supplementary Figure S4**

**Figure S4: (A)** Correlation of overall survival of AML patients to CD86 and PD-L1 expression. **(B)** Expression of various human (CD80, CD86, PD-L1, CD137, CD137L, CD40, CD40L, CD28, PD-1, TIM-3, CTLA-4) and mouse (CD80, CD86, PD-L1) antigens on the Ba/F3 cell line.

### Supplementary Tables

Table S1: Characteristics of AML patients used for *ex vivo* evaluation

| **Patient** | **Sex** | **Age** | **Diagnosis** | **% blasts** | **Cytogenetics** | **FLT3-ITD mut** | **NPM1 mut** |
| --- | --- | --- | --- | --- | --- | --- | --- |
| 1 | f | 21 | FD | 50 | 45 X,−X, t(8;21)(q22;q22)/ 46, XX | − | − |
| 2 | f | 57 | FD | 94 | 46, XX | − | + |
| 3 | f | 69 | FD | 84 | 46, XX | + | - |
| 4 | f | 80 | FD | unknown | unknown | unknown | unknown |
| 5 | f | 21 | FD | 88 | 46, XX | + | + |
| 6 | m | 84 | FD | 47 | 46, XY | − | + |
| 7 | m | 82 | FD | 70 | 46, XY, complex-aberrant | - | - |
| 8 | m | 72 | FD | 57 | 46, XY | - | + |
| 9 | m | 76 | FD | 84 | 46, XY, der(4)t(4;7)(q31;q31),der(7)t(4;7)(q31;q22) | - | - |
| 10 | f | 66 | FD | 68 | 46, XX, inv(16)(p13q22)/46, XX | - | - |
| 11 | m | 44 | FD | 80 | 46, XY, inv(16)(p13q22) | - | - |
| 12 | m | 62 | FD | 94 | 47, XY, +8/ 46, XY | + | + |
| 13 | m | 44 | FD | 70 | 46, XY, inv(16)(p13q22) | - | - |
| 14 | f | 83 | FD | 70 | unknown | - | + |

FD: first diagnosis; ITD: internal tandem duplication.

Table S2: Antibodies used for flow cytometry

| **Antigen (human)** | **Fluorochrome** | **Clone** | **Manufacturer** |
| --- | --- | --- | --- |
| CD2 | BV421 | TS1/8 | Biolegend (30921) |
| CD2 | BV605 | IV T085 | Biolegend (300224) |
| CD3 | APC/Cy7 | UCHT1 | Biolegend (300426) |
| CD11a/CD18 (LFA-1) | PE | m24 | Biolegend (363406) |
| CD28 | FITC | CD28.2 | Biolegend (302906) |
| CD33 | PE | WM53 | Biolegend (303404) |
| CD33 | APC | WM53 | Biolegend (303408) |
| CD33 | PerCP/Cy5.5 | WM53 | Biolegend (303414) |
| CD33 | PE/Cy7 | WM53 | Invitrogen (25-0338-42) |
| CD40 | PE | 5C3 | eBiosciences (12-0409-42) |
| CD40L | BV421 | 24-31 | Biolegend (310824) |
| CD45 | Krome Orange | J33 | Beckman Coulter (B36294) |
| CD45 | PE/Cy7 | HI30 | Biolegend (304014) |
| CD45RA | BV421 | HI100 | Biolegend (304130) |
| CD80 | APC | 2D10 | Biolegend (305220) |
| CD86 | BD Horizon™ V450 | 2331(FUN-1) | BD Biosciences (560357) |
| CD86 | PE-CF594 | 2331(FUN-1) | BD Biosciences (562390) |
| CD86 | Pacific Blue | IT2.2 | Biolegend (305423) |
| CD86 | PE | IT2.2 | Biolegend (305406) |
| CD137 | PE/Cy7 | 4B4-1 | Biolegend (309818) |
| CD137L | PE | 5F4 | Biolegend (311504) |
| CD152 | PE | 14D3 | Invitrogen (12-1529-42) |
| CD197 (CCR7) | PE | G043H7 | Biolegend (353204) |
| CD274 | PE | 29E.2A3 | Biolegend (329705) |
| CD274 | FITC | MIH1 | BD Biosciences (558065) |
| CD274 | PE | MIH1 | BD Biosciences (557924) |
| CD274 | Pe/Cy7 | MIH1 | BD Biosciences (558017) |
| CD274 | APC | MIH1 | Invitrogen (17-5983-42) |
| CD279 | BV785 | NAT105 | Biolegend (367432) |
| CD366 | BV421 | F38-2E2 | Biolegend (345008) |
| GZMB | PerCP-Cy5.5 | QA16A02 | Biolegend (309218) |
| GZMB | Pacific Blue | GB11 | Biolegend (515408) |
| pERK1/2-pT202/pY204 | AF647 | 20A | BD Biosciences (612593) |
| pAkt-pS473 | PE | M89-61 | BD Biosciences (560378) |
| pZAP70-pY349/Syk (Y352) | Pe/Cy7 | 17A/P-ZAP70 | BD Biosciences (561458) |
| **Antigen (mouse)** |  |  |  |
| CD80 | APC | 16-10A1 | Biolegend (104713) |
| CD86 | PE | GL-1 | Biolegend (105007) |
| CD274 | FITC | MIH-6 | Bio-Rad (MCA2626F) |
| F4/80 | BV421 | BM8 | Biolegend (123132) |
